# TSLP is differentially regulated by vitamin D3 and cytokines in human skin

**DOI:** 10.1002/iid3.48

**Published:** 2015-02-16

**Authors:** Janneke Landheer, Barbara Giovannone, Svetlana Sadekova, Sandra Tjabringa, Claudia Hofstra, Koen Dechering, Carla Bruijnzeel-Koomen, Charlie Chang, Yu Ying, Rene de Waal Malefyt, DirkJan Hijnen, Edward Knol

**Affiliations:** 1 Department of Dermatology & Allergology, University Medical Center UtrechtUtrecht, the Netherlands; 2 Biologics Discovery, Merck Research LaboratoriesPalo Alto, California; 3 Department of Immunology, Merck Sharpe and DohmeOss, the Netherlands; 4 Information Technology, Merck Research LaboratoriesPalo Alto, California; 5 Department of Immunology, Merck Research LaboratoriesPalo Alto, California; 6 Department of Immunology, University Medical Center UtrechtUtrecht, the Netherlands

**Keywords:** Allergy, atopic dermatitis, TSLP, VDRE, vitamin D3

## Abstract

Thymic stromal lymphopoietin (TSLP) plays an important role in allergic diseases and is highly expressed in keratinocytes in human lesional atopic dermatitis (AD) skin. In nonlesional AD skin TSLP expression can be induced by applying house dust mite allergen onto the skin in the atopy patch test. Several studies have demonstrated that the induction of TSLP expression in mouse skin does not only lead to AD-like inflammation of the skin, but also predisposes to severe inflammation of the airways. In mice, TSLP expression can be induced by application of the 1,25-dihydroxyvitamin D3 (VD3) analogue calcipotriol and results in the development of eczema-like lesions. The objective is to investigate the effect of VD3 (calcitriol) or calcipotriol on TSLP expression in normal human skin and skin from AD patients. Using multiple ex vivo experimental setups, the effects of calci(po)triol on TSLP expression by normal human skin, and skin from AD patients were investigated and compared to effects of calcipotriol on mouse and non-human primates (NHP) skin. No induction of TSLP expression (mRNA or protein) was observed in human keratinocytes, normal human skin, nonlesional AD skin, or NHP skin samples after stimulation with calcipotriol or topical application of calcitriol. The biological activity of calci(po)triol in human skin samples was demonstrated by the increased expression of the VD3-responsive Cyp24a1 gene. TSLP expression was induced by cytokines (IL-4, IL-13, and TNF-α) in skin samples from all three species. In contrast to the findings in human and NHP, a consistent increase in TSLP expression was confirmed in mouse skin biopsies after stimulation with calcipotriol. VD3 failed to induce expression of TSLP in human or monkey skin in contrast to mouse, implicating careful extrapolation of this often-used mouse model to AD patients.

## Introduction

Atopic dermatitis (AD) is a common chronic inflammatory skin disease. The pathogenesis of AD has been associated with both skin barrier defects and immune response abnormalities.

Thymic stromal lymphopoietin (TSLP) has been shown to play an important role in the initiation and maintenance of the allergic immune response. TSLP polymorphisms in humans have been linked to increased disease risk for AD, asthma, and allergic rhinitis 1–3. TSLP is abundantly expressed by keratinocytes in lesional AD skin, but not in nonlesional AD skin or normal skin from healthy donors 4. In nonlesional AD skin TSLP expression can be induced by applying house dust mite allergen onto the skin in the atopy patch test 5. The inflammatory cytokines IL-4, IL-13, and TNF-α as well as microbes, and mechanical injury induce TSLP expression in normal skin 6–11. Fibroblasts, smooth muscle cells, mast cells, and basophils are also able to produce TSLP in the skin following activation 4,12. TSLP acts on various cell types including dendritic cells (DC), mast cells, and T helper cells. TSLP-activated DCs prime naive T cells towards a Th2 phenotype, producing cytokines involved in allergic inflammation including IL-4, IL-5, IL-13, and TNF-α 4. In addition, TSLP-activated-DCs have been shown to produce thymus and activation-regulated chemokine (TARC/CCL17) and macrophage-derived chemokine (MDC/CCL22) 4. Recently, TSLP has been shown to directly activate sensory neurons and induce itch, an important characteristic of AD 13. Approximately two thirds of all AD patients develop allergic rhinitis and half develop allergic asthma later in life 14. This suggests that AD is a starting condition, which increases the likelihood of developing other allergic diseases, a phenomenon also known as the atopic march. Several studies have demonstrated that the induction of TSLP expression in mouse skin does not only lead to AD-like inflammation of the skin, but also predisposes to severe inflammation of the airways 15–19. For example, Han et al. demonstrated that allergen-induced TSLP expression in skin selectively leads to severe CD4^+^ T cell-driven inflammation in the airways 16. TSLP may therefore play a central role not only in the development of AD, but also in the development of other allergic diseases.

There is a growing body of evidence that VD3 is an important regulator of cutaneous immunity. VD3 has been shown to increase cathelicidin expression and antimicrobial activity in keratinocytes in vivo and in vitro^20^–^22^. VD3 either alone or in combination with cytokines induces FOXP3-expressing regulatory T cells in vitro 23,24. VD3 and/or its receptor also play important roles in barrier function by the inhibition of proliferation and stimulation of differentiation of keratinocytes 25. Moreover, VD3 analogues are successfully used in the treatment of psoriasis, an inflammatory skin disease characterized by a predominant Th17 cell infiltrate and hyperproliferative keratinocytes.

In mice, epidermal TSLP production can be induced by topical application of 1,25-dihydroxy-vitamin D3 (VD3) and its synthetic analogue calcipotriol (or MC903), resulting in an AD-like phenotype characterized by eczematous lesions with xerosis and pruritis, skin infiltrates of CD4+ Th2 T cells, DC, eosinophils and mast cells. In addition, increased expression of inflammatory cytokines (IL-4, IL-5, IL-13, IL-31, IFN-γ) and elevated serum levels of IgG and IgE were observed 12. Despite extensive work on the effect of VD3 on TSLP expression in murine models, very limited data is available on its effect in human skin.

The purpose of the current research was to examine the effect of VD3 on TSLP expression in normal human skin and skin from AD patients. The effects of VD3 were studied using different experimental designs. In addition, mouse and monkey skin were stimulated ex vivo with VD3 to determine a possible broader species-specific effect of VD3 on TSLP expression.

## Methods

### Human primary keratinocytes culture

Skin from normal adults was obtained as resected tissue following surgical procedures. Human primary adult keratinocytes were isolated from normal skin as described previously 26. Passage 2 keratinocytes were cultured in keratinocyte growth medium (KGM-2) (Lonza Group Ltd., Basel, Switzerland) until 70% confluence and differentiated for three days in the same medium, supplemented with calcium up to 1.3 mM. Cells were differentiated for two more days in hydrocortisone-depleted KGM-2 with calcium, since hydrocortisone has been described to decrease TSLP expression 9,10. Cells were stimulated with 100 nM calcipotriol (Sigma–Aldrich Co., St Louis, MO) in DMSO (0.1%), DMSO 0.1% (Sigma–Aldrich) or a cytokine mixture containing IL-4 (100 ng/mL), IL-13 (100 ng/mL), and TNF-α (20 ng/mL) (R&D Systems, Minneapolis, MN), as positive control for the induction of TSLP [E(8)]. A calcipotriol concentration curve was performed at 10, 30, 100, and 300 nM (*n* = 1).

### Normal human skin biopsies culture

Normal human skin biopsies were obtained as resected material after cosmetic surgery procedures. Four millimeter biopsies (*n* = 3) were cultured in Dulbecco's Modified Eagle Medium (DMEM) containing 10% FBS, 1% pyruvate, 1% HEPES, and 2% penicillin/streptomycin and stimulated with 100 nM calcipotriol, DMSO, or a cytokine mixture (see above). A calcipotriol concentration curve was performed at 10, 30, 100, and 300 nM (*n* = 1). To study the effects of a topically applied commercially available VD3 ointment (calcitriol; Silkis®, Galderma, Switzerland), calcitriol ointment or petrolatum were applied to a 20 cm^2^ piece of full thickness (including subcutis) normal human skin (*n* = 3), for 30 min at 37 °C. One third of the skin surface was left untreated and was used as control. After incubation, 4 mm biopsies were taken and cultured at air-liquid interface in the medium described above on a trans-well system (Greiner Bio-One, Germany) for 24 h, 48 h, and 72 h. Culture media level was adjusted to ensure optimal air exposure of the epidermis.

### Human atopic dermatitis skin biopsies

Six adult AD patients (diagnosis according to the Hanifin and Rajka criteria 27;18–70 years old) with mild to moderate eczema, using topical treatment only, were recruited from the outpatient clinic. All patients had IgE specific to house dust mite (determined by CAP or ISAC) and a positive atopy patch test to house dust mite. Patients had a mean age of 28 years; 83% were female; mean SCORAD score was 11. All patients provided written informed consent before study enrolment. The study was approved by the institutional medical ethical committee and experimental procedures were performed according to the Declaration of Helsinki principles.

Three 4 mm biopsies were taken from nonlesional back skin, which had not been exposed to UV-light or topical corticosteroids for at least two weeks prior to inclusion. The biopsies were cultured submerged in 100 nM calcipotriol, DMSO (0.1%) or cytokines mixture (see above). After 48 h of culture, supernatants were collected and biopsies were embedded in Tissuetek and stored at −80 °C until further analysis.

### Mouse & monkey skin

Ear and back skin from wild type BALB/c mice (Taconic, Germantown, NY) was used for mouse skin experiments. Ear skin was split with forceps into dorsal and ventral halves prior to culture. Mice were maintained in an AAALAC accredited facility according to the “Guide for the Care and Use of Laboratory Animals” 8th edition (2011) prepared by the Institute of Laboratory Animal Resources, National Research Council. Animal protocols were approved by the Institutional Animal Care and Use Committee of Merck Research Labs, Palo Alto. *Macaca fascicularis* (or Cynomolgus monkeys [cyno]) were purchased from Alphagenesis (Yemassee, SC) and maintained at East Carolina University (ECU), Brody School of Medicine, Greenville, NC. Animal husbandry was conducted according to US Department of Agriculture guidelines and according to the “Guide for the Care and Use of Laboratory Animals” 8th edition (2011) (see above) and Animal Welfare Act regulations. Animal protocols were approved by the Institutional Animal Care and Use Committee of East Carolina University (Greenville, NC). Cyno back skin samples were collected at euthanasia. Veterinary care was given to animals requiring medical attention.

After subcutaneous fat removal, skin specimens were cut into small (approximately 2–3 mm) pieces and cultured at air-liquid interface in DMEM supplemented with 4.5 g/L glucose, 1% L-glutamine, 0.11 g/L sodium pyruvate, 10% FBS, 100 u/mL penicillin, 100 ug/mL streptomycin, and 40 ug/mL gentamycin. Skin specimens were stimulated for 24 h (mouse) or 48 h (cyno) with cytokines (IL-4 [100 ng/mL], IL-13 [100 ng/mL], TNF-α [20 ng/mL] [R&D Systems, Minneapolis, MN]), or (mouse: 10 and) 100 nM calcipotriol in ethanol (Tocris Bioscience, Minneapolis, MN). Culture supernatants and skin samples were collected and frozen at −80 °C.

### Real-time PCR

*Human material*: Total RNA was isolated from biopsy-derived cryosections (60 × 20 µm cryosections), using RNeasy Micro kit (Qiagen N.V., Venlo, The Netherlands). RNeasy Mini kit (Qiagen) was used to isolate total RNA from keratinocyte cultures. cDNA synthesis was performed using a BioRad iScript cDNA synthesis kit (BioRad, Hercules, CA). Real-time quantitative PCR was done on a BioRad MyiQ real time PCR Detection System, using iQ SYBR Green Supermix kit (BioRad). Two forms of TSLP mRNA (long and short) have been described in literature, with only the long transcript is translated into a functional protein 2,28. A primers set which specifically detects the long form TSLP transcript was designed. The following primers were used: TSLP forward: AGTGGGACCAAAAGTACCGAGTT, TSLP reverse: GGATTGAAGGTTAGGCTCTGG, Cyp24 forward: GGTGACATCTACGGCGTACAC, CYP24 reverse: CTTGAGACCCCCTTTCCAGAG and glyceraldehyde 3-phosphate dehydrogenase (GAPDH) forward: AGAAGGCTGGGGCTCATTT, GAPDH reverse: GAGGCATTGCTGATGATCTTG. The amount of each mRNA was normalized to the amount of GAPDH in the same sample. Relative increases in mRNA expression were calculated using the 2^-ΔΔCT method 29.

*Mouse and monkey material*: Frozen skin samples were homogenized using Cryogenic Tissue Pulverizer (Research Products Int., Mount Prospect, IL). Total RNA was isolated using RNeasy Midi kit (Qiagen, Chatsworth, CA) according to the manufacturer's instructions. RNA quality was assessed by Agilent Bioanalyzer Nanochip (Agilent Technologies, Santa Clara, CA) analysis to check for intact 28 S and 18 S ribosomal RNA bands.

Total RNA was treated with DNase I to remove any contaminating genomic DNA (Ambion, Austin, TX), then reverse transcribed into cDNA using a combination of random hexamers and oligo-dT primers (Promega, Madison, WI). Gene expression levels were measured using real-time quantitative PCR and the ABI 7300 Sequence Detection System (Perkin Elmer, Applied Biosystems, Foster City, CA). Primers for human Cyp24 that crossreact to cyno Cyp24 were obtained commercially from Applied Biosystems (Foster City, CA). Real-time PCR amplification of the housekeeping gene ubiquitin was performed for each sample to allow for normalization between samples by the Δ-Δ Ct method (ABI User Bulletin #2, 1997). The equation 1.8 e (Ct of ubiquitin minus Ct of gene being measured) × 104 was used to obtain normalized values. The Δ-Δ Ct method described above results in normalized expression values relative to the housekeeping gene ubiquitin. Normalized values less than 1.0 are considered to be at the limit of detection for this method and were considered to be negative for analysis.

### TSLP ELISA

Human TSLP protein concentrations in cell-free culture supernatants were quantified with a human TSLP-specific ELISA kit (Biolegend, San Diego, CA). For TSLP protein detection in mouse cell-free culture supernatants, a mouse TSLP-specific ELISA kit was used (R&D Systems). Cyno TSLP was detected using in-house Assay for Cynomolgus Monkey TSLP Baseline using an electrochemiluminescence (ECL) immunoassay method. Briefly, a sandwich is formed on the MSD plate (Meso Scale Discovery, Rockville, MD) surface between cyno TSLP in the sample and both capture reagent and detection antibody. The capture reagent is a biotinylated rat anti-human TSLP monoclonal antibody GNE01.23B12.H8.A4. A rat anti-NHP TSLP monoclonal antibody, labeled with ruthenium tris-bipyridine chelate Rat anti [TSLP_NHP] JL10.34H11.A8, is used as the detection antibody. This antigen-antibody complex is captured by streptavidin, which is coated on the MSD plate surface. The plate is put into the Meso Scale Discovery SECTOR™ Imager 6000 and a voltage is applied to the bottom of the plate initiating the ECL signal from the label upon electrochemical stimulation at the electrode surface. The resulting signal produced is directly proportional to the concentration of cyno TSLP in the sample.

### Statistical analyses

Data were analyzed using the Statistical Package for the Social Sciences Software, SPSS for Windows, Version 15.0 (SPSS Inc., Chicago, IL). For human skin data, significance of increase in gene or protein expression was calculated with a one-sample t-test, using 1 or 0 as reference values, respectively. For cyno skin data, significance of increase in gene and protein expression (relative to control condition) was computed using Wilcoxon's matched-pairs signed rank test. P-values <0.05 were considered significant.

## Results

### VD3 analogue calcipotriol does not induce TSLP in primary human keratinocytes and SCC lines

**Figure 1:**
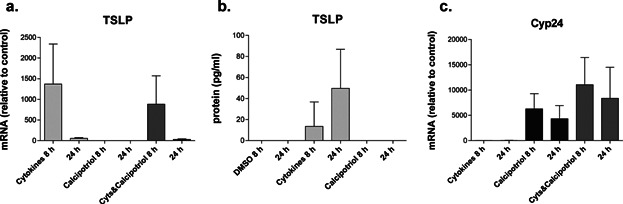
Effect of calcipotriol on differentiated primary human keratinocytes. Differentiated keratinocytes were stimulated with calcipotriol alone, a mix of cytokines (IL-13,IL-4, TNF-α) (Cyts) and a combination of the two treatments for 8 or 24 h. (a) TSLP mRNA expression (*n* = 4); (b) TSLP protein expression (*n* = 3); (c) Cyp24 mRNA expression (*n* = 4). The control for cytokines stimulated cells is medium, in the other conditions the control is DMSO. Data are shown as mean +/− SEM.

To study the effects of calcipotriol on TSLP expression by human keratinocytes, differentiated primary adult keratinocytes were stimulated for 8 and 24 h with calcipotriol (100 nM) and TSLP gene and protein expression were analyzed. A cytokine mixture of IL-4 (100 ng/mL), IL-13 (100 ng/mL), and TNF-α (20 ng/mL), which was shown to induce TSLP expression in keratinocytes and skin biopsies, was included as control 8. Only the long form TSLP transcript was measured, since it was demonstrated that only the long form of TSLP is translated into a functional TSLP protein and can be effectively induced by many stimuli 2,28,30. Calcipotriol-stimulated keratinocytes did not show an increase in TSLP gene expression, while the cytokine mixture did, even at an early time point (Fig. 1a). Only cytokine-stimulated keratinocytes showed increased expression of TSLP protein release after 8 and 24 h (Fig. 1b) as measured by ELISA. The biological activity of calcipotriol was confirmed by the strong induction of VD3 24-hydroxylase (*Cyp24a1*) gene expression (Fig. 1c) 12. To investigate if VD3 enhances cytokine-induced TSLP expression, which may more closely resemble the physiological situation, keratinocytes were stimulated with both calcipotriol and cytokines for 8 and 24 h. The addition of calcipotriol did not affect cytokine-induced TSLP expression (Fig. 1a). In differentiated keratinocytes, cytokine-induced TSLP gene expression was 10–20 fold higher than in undifferentiated keratinocytes. Baseline VDR expression levels were similar in differentiated and undifferentiated keratinocytes (data not shown). Limited data are available on the induction of TSLP by VD3 in humans. TSLP was identified in a large-scale in silico and microarray study as a potential direct VD3 target in the human squamous carcinoma cell line SCC25 31. Since this result was not validated at the protein level and the expression of the long or short form of TSLP was not addressed, we tested SSC-25 and several other human squamous carcinoma cell lines (SCC-4, SCC-9, SCC-25, CAL-27) for calcipotriol responsiveness. Calcipotriol at 100 nM did induce *Cyp24a1* mRNA but not TSLP mRNA or protein in any of the cell lines (data not shown).

### No TSLP production by calcipotriol-stimulated normal human skin biopsies

**Figure 2:**
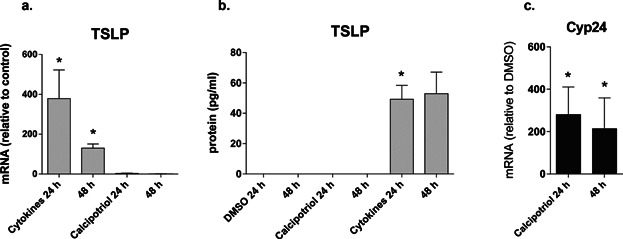
Effects of calcipotriol on TSLP expression in normal human skin biopsies at 24 and 48 h of treatment. (a) TSLP mRNA expression; (b) TSLP protein expression; (c) Cyp24 gene expression. Data are representative of three independent skin donors and are shown as mean ± SEM.; *P < 0.05.

The results above demonstrated that isolated primary human keratinocytes do not show induction of TSLP expression in response to VD3. However, dermal fibroblasts, mast cells and basophils are also able to produce TSLP in skin 4,12. Therefore, it was important to study TSLP expression in calcipotriol-stimulated intact normal human skin biopsies, which retain all the different cell types and structures present in human skin. When punch biopsies were cultured submerged for 24 or 48 h in calcipotriol (100 nM)-containing medium, no increase in TSLP mRNA (Fig. 2a) or protein (Fig. 2b) levels was observed. However, the IL-4, IL-13, and TNF-α combination was able to induce TSLP gene expression and protein release (Fig. 2a, b). Increased *Cyp24a1* expression confirmed the biological activity of calcipotriol (Fig. 2c).

### Topical calcitriol ointment application does not affect TSLP expression in normal human skin biopsies

**Figure 3:**
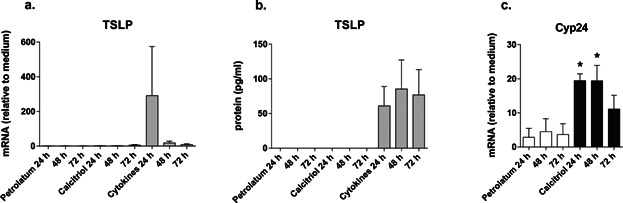
Effects of topical application of calcitriol on TSLP expression in normal human skin samples. Biopsies were cultured in an air-liquid interphase manner for 24, 48, and 72 h post topical application of calcitriol and petrolatum control (30 min). Cytokines mixture was added to the culture medium of untreated skin biopsies as positive control for TSLP stimulation. (a) TSLP mRNA expression; (b) TSLP protein expression; (c) Cyp24 expression. Data are representative of three independent skin donors and are shown as mean ± SEM.; *P <  0.05.

Topical application of VD3 or calcitriol ointment is a well-established treatment for psoriasis and has recently been shown to induce TSLP expression in lesional psoriasis skin 32. To study the effects of topically applied calcitriol on normal human skin, biopsies were collected after topical calcitriol application and cultured at air-liquid interface for 24, 48, and 72 h. *Cyp24a1* expression confirmed the biological activity of the VD3 ointment (Fig. 3c). Neither TSLP mRNA induction nor TSLP protein release were observed after application of calcitriol, while the cytokine mixture applied in the culture induced both TSLP gene and protein expression (Fig. 3a, b).

### No TSLP production by calcipotriol-stimulated nonlesional human AD skin biopsies

**Figure 4:**
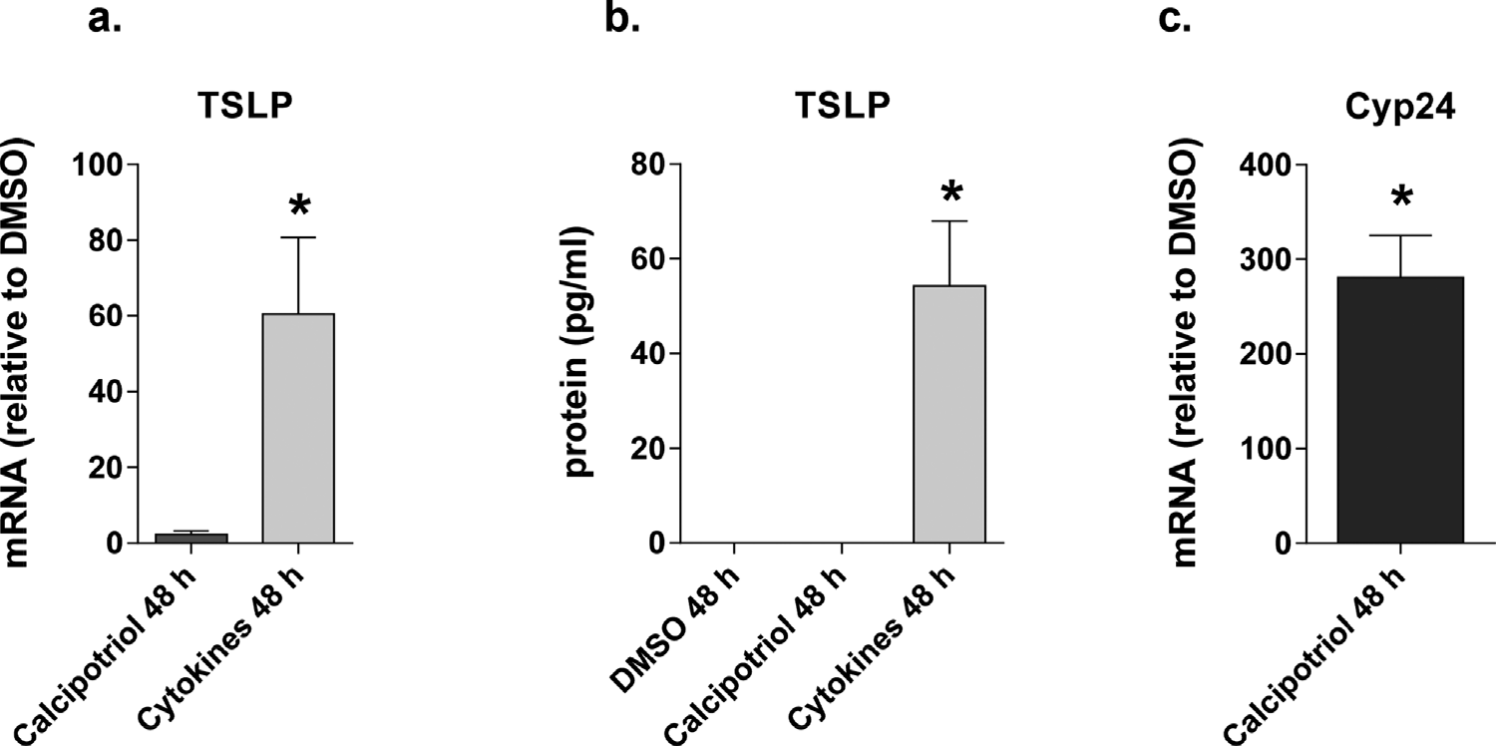
Effects of calcipotriol on nonlesional AD skin biopsies (*n* = 6) stimulated for 48 h. (a) TSLP mRNA expression; (b) TSLP protein expression; (c) Cyp24 mRNA expression. Data are shown as mean ± SEM.; *P < 0.05.

Nonlesional AD skin can be regarded as immunologically pre-activated, showing hyperproliferative keratinocytes and increased numbers of T cells and inflammatory cytokines 33–37. Even though calci(po)triol did not induce TSLP expression in normal skin, it could be possible that VD3 is able to increase TSLP expression in immunologically activated nonlesional AD skin. Therefore, nonlesional AD skin biopsies were stimulated for 48 h. Baseline *TSLP* gene expression (relative to GAPDH) in unstimulated human skin biopsies was similar in NL AD skin (*n* = 6; mean 16.0; SD 0.8) and healthy control skin (*n* = 3; mean 16.5; SD 1.3). The cytokine mixture induced TSLP gene expression and protein release, however, TSLP expression was unaffected by calcipotriol exposure (Fig. 4a, b). The biological activity of calcipotriol was confirmed by the increased *Cyp24a1* expression (Fig. 4c).

### VD3 analogue calcipotriol induces TSLP expression in mouse skin in vitro

**Figure 5:**
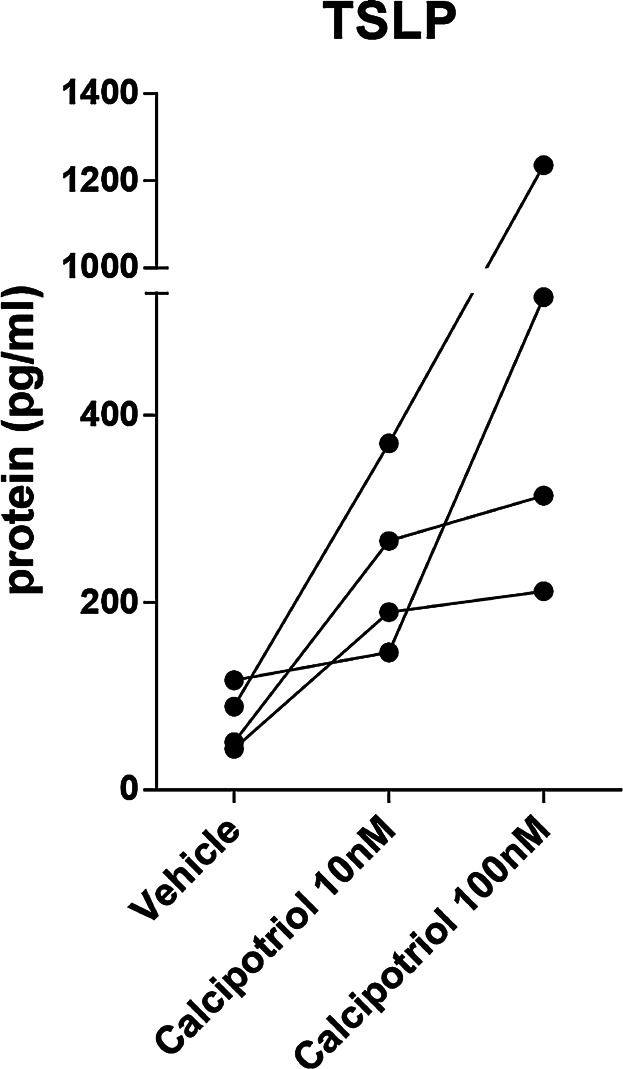
Calcipotriol induces TSLP protein expression in mouse skin biopsies (*n* = 4) after 24 h stimulation. Each dot represents TSLP levels in a culture of six biopsies per mouse.

VD3 has previously been shown to induce TSLP expression in mouse skin in vivo 12. To confirm TSLP upregulation in mouse skin in vitro, and to correlate results to our findings in human skin ex vivo, mouse skin biopsies were cultured in a similar setting described above. Biopsies were stimulated with calcipotriol (10 and 100 nM) in ethanol, or vehicle alone (ethanol) (Fig. 5) for 24 h in air-exposed cultures. Six biopsies per mouse were pooled in one well. In cytokines-stimulated mouse skin samples, TSLP protein expression increased from undetectable at baseline to 0–1,116 and 3,531–4,495 pg/mL after 24 and 48 h stimulation, respectively. Calcipotriol-stimulated skin samples consistently showed increased expression of TSLP protein (Fig. 5), at both doses tested. The failure of VD3 to induce TSLP expression in all examined human skin cultures was therefore not due to the experimental design and conditions, but may be a species-related phenomenon.

### Calcipotriol does not induce TSLP expression in Cynomolgus monkey skin

**Figure 6:**
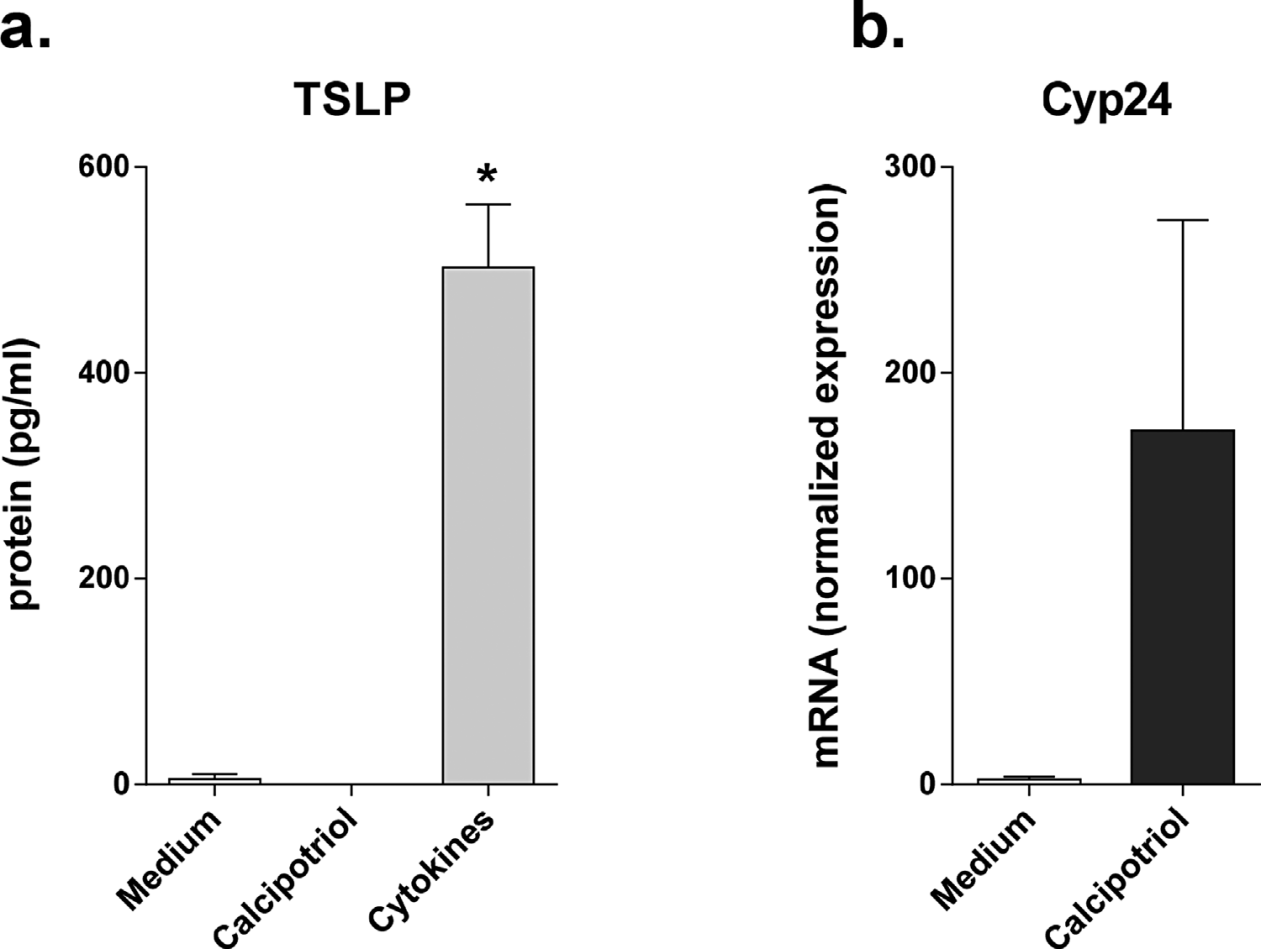
Effects of the VD3 analogue calcipotriol on Cynomolgus monkey skin biopsies. (a) TSLP protein expression (*n* = 4) and (b) Cyp24 mRNA expression (*n* = 3), after 48 h stimulation. Data are shown as mean ± SEM; *P < 0.05.

To test the hypothesis that VD3 does not induce TSLP expression in NHP skin, Cynomolgus monkey skin biopsies were stimulated with calcipotriol (100 nM) for 48 h. Similar to our observation in human samples, VD3 treatment did not result in increased TSLP protein release, while cytokines stimulation did induce TSLP production (Fig. 6a). The biological activity of calcipotriol was confirmed by the increased *Cyp24a1* expression (Fig. 6b).

## Discussion

The current study shows that VD3 or its analogues (VD3As) could not induce TSLP expression in primary human keratinocytes, normal human skin, human AD skin, and normal monkey skin. In contrast, using identical protocols, VD3A induced TSLP expression in ex vivo mouse skin cultures supporting previous observations that application of the physiologically active VDR ligand VD3 (calcitriol) or its low-calcemic analogue (calcipotriol) on mouse skin induces TSLP expression by epidermal keratinocytes in vivo 12. Altogether, these results indicate that VD3 differentially affects the expression of TSLP in murine versus human and non-human primate skin. Failure to detect upregulation of TSLP by VD3 was not related to VD3 concentration or route of administration. Several experimental designs and different modes of administration of VD3A were employed to investigate the effects of VD3A on TSLP expression in human skin. The effects of VD3A on primary human keratinocytes and biopsies from human healthy control skin were investigated at concentrations similar to the ones used in mouse experiments (10 and 100 nM). TSLP induction was not detected, even when the concentration of VD3A was increased up to 300 nM (data not shown). In agreement with these data, it was recently shown that in primary human keratinocytes high levels of VD3A (up to 10 µM) induced increased gene expression of total TSLP, but did not affect the long form TSLP transcript, and did not result in protein release 30. In addition to a different source of primary keratinocytes and an alternative method of differentiation, our data expand on those findings by Xie at al., confirming the absence of any additional induction of TSLP in primary human keratinocytes after adding VD3A to cytokines. Moreover, our data from squamous carcinoma cell lines showed absence of TSLP upregulation after VD3 stimulation, indicating that neither our data nor published data show that the long form of TSLP mRNA is responsive to VD3 in human skin.

Failure to detect upregulation of TSLP by VD3 was also not related to interference by the microenvironment or pathology. VD3 could affect not only keratinocytes, but also inflammatory cells, including dendritic cells, macrophages, and T cells. Therefore, the interaction between inflammatory cells and keratinocytes could be important in the VD3-induced regulation of TSLP expression in human skin. Residual TSLP expression was found in mast cells, basophils and/or fibroblasts after keratinocyte-selective TSLP ablation in a VD3-induced eczema mouse model 4,12. Thus, to preserve the skin microenvironment in assessing VD3A-responsivenesss, our ex vivo experiments examined the effect of VD3A in a physiological setting using skin biopsies. Even in these experiments no expression of TSLP was found after culture with calcipotriol at 10–300 nM, or following topical treatment with calcitriol at its common therapeutic concentration of 100 nM (Fig. 3 and data not shown) 32. Thus, VD3 failed to induce TSLP when the skin microenvironment was preserved.

Nonlesional AD skin is different from normal skin and it is possible that the “activated” state of nonlesional AD skin may facilitate TSLP expression after VD3-stimulation. In AD there is impairment of the skin barrier, and keratinocytes have been found to express decreased amounts of skin barrier proteins, such as filaggrin, loricrin, and involucrin, which may result in increased permeability to allergens and topically administered drugs 38. Furthermore, keratinocytes from nonlesional AD skin express increased levels of GM-CSF in vitro, and show increased proliferation compared to normal keratinocytes 36. In addition, compared to normal skin, the number of tissue-resident T cells is increased in nonlesional AD skin and T cell-derived cytokines (including IL-4, IL-13, TNF-α) are expressed that can induce TSLP expression in keratinocytes. However, application of VD3A did not affect TSLP expression in nonlesional AD skin indicating that nonlesional AD skin is not predisposed to VD3 responsiveness. In psoriasis, topical application of VD3 results in enhanced differentiation and decreased proliferation of keratinocytes and induction of regulatory T cells 39. Sato-Deguchi et al. recently showed increased TSLP protein expression in lesional psoriasis skin following topical VD3As application 40. However, in their experiments biopsies were examined from patients that were treated with VD3As for periods of 1 month to 1 year in combination with topical steroids. It is thus possible that induction of TSLP by this chronic administration of VD3As is not a direct effect of VD3As but due to secondary effects of this treatment.

A possible explanation for the difference in TSLP induction by VD3 in mouse versus human and monkey could reside in the regulatory elements of the TSLP promoter. Li et al. hypothesized that VD3-induced TSLP expression results from the binding of the VD3/VDR/RXR-complex to putative VD3 response elements (VDREs) in the murine TSLP promoter sequence 12. In this model, in the basal condition with no RA and little or absent active VD3 around, the TSLP promoter is silenced by unligated RXRa(b)/VDR and RXRa(b)/RAR heterodimers associated with corepressors. The binding of RA and/or VD3 releases the repression signals and allows TSLP transcription. RA(retinoic acid)g/RAR/RXR-complexes bound to RA response elements (RAREs) act synergistically with VDRE-bound VD3/VDR/RXR-complexes in induction of TSLP expression. Since VDR, RAR, and RXR expression are required for TSLP induction 12, we examined mRNA expression of VDR and RAR in human and mouse skin and confirmed the expression of various isoforms, as expected by the observed *Cyp24a1* responsiveness in our experiments (data not shown). The observed absence of induction of TSLP by VD3 suggests that the RXR/VDR-coactivator complexes induced by VDRAs in humans may not be able to relieve the repression exerted by RAR/RXR (a). As alternative explanation, the architecture of the VDREs (DR3) and RAREs (DR1, DR2) in the human TSLP promoter may be too different from those in the mouse promoter to allow predictions from the mouse model of TSLP RXR/VDR/RAR-mediated transcriptional regulation to be extrapolated to human. A preliminary analysis in silico showed that the VDRE DR3b is proximal to the RAREs in the human promoter and the directionality of DR1, DR3a, and DR3b are opposite in mouse and human (Table1). When compared in silico, the location and the sequences of putative specific VDREs and RAREs in the TSLP promoters of Cynomolgus and Rhesus Macaques and humans were conserved. Thus, the difference in promoter elements between mice and primates could explain the different outcomes after VD3 and VD3A stimulation of the skin. This is a preliminary observation based on an in silico analysis and its validation will require additional genetic and biochemical studies to determine the ability of the TSLP promoter to respond to the VD3 in both human and NHP.

**Table 1:** Identification of VDRE and RARE elements in the promoters of mouse, cynomolgus monkey, rhesus monkey and human TSLP. Indicated are genome version of the database, search pattern, orientation and location relative to the start codon

Species	Gene	Chr#	Genome version	Genome start	Genome end	Binding element for	Pattern	Seq found	Seq strand	Seq start	Seq end	Seq start relative to start-codon
Mouse	Tslp	18	mm9 July 2007	32955037	32979453	DR3a	AGGACAgccAGGGCT	AGGACAGCCAGGGCT	Direct	6360	6374	−13658
						DR3b	GAGCCAgagGGGTCA	GAGCCAGAGGGGTCA	Reverse	12649	12663	−7369
						DR3c	AGGACAgccAGGGCT	AGGACAGCCAGGGCT	Direct	13512	13526	−6506
						DR3d	AGGACAgccAGGGCT	AGGACAGCCAGGGCT	Direct	15797	15811	−4221
						DR1	GGGTCAgGGGACA	GGGTCAGGGGACA	Direct	17842	17854	−2176
						DR2	AGCTCAacAGGTCA	AGCTCAACAGGTCA	Direct	18955	18968	−1063
Human	TSLP	5	GRCh37	110387390	110413722	DR3a	AGTTCTaaaGGTTCA	AGTTCTAAAGGTTCA	Reverse	13167	13181	−7033
						DR2	AGGACAatGGGTAT	AGGACAATGGGTAT	Direct	15018	15031	−5182
						DR1	AGGACAcAAGTCA	AGGACACAAGTCA	Reverse	16140	16152	−4060
						DR3b	AG[gc]ATAatgAGGTCA	AGGATAATGAGGTCA	Direct	17544	17558	−2656
Rhesus	TSLP	6	rheMac2	107356392	107370573	DR3a	AGTTCTaaaG[G^*^]TTCA	AGTTCTAAAGTTCA	Reverse	3006	3019	−7023
						DR2	AGGACAatGGGTAT	AGGACAATGGGTAT	Direct	4851	4864	−5178
						DR1	AGGACAcAAGTCA	AGGACACAAGTCA	Reverse	5973	5985	−4056
						DR3b	[at]G[agc][at][tc][ac]a[at]gAGGTCA	TGATCCAAGAGGTCA	Direct	7639	7653	−2390
Cyno	TSLP	6	macFas1	109349628	109363897	DR3a	AGTTCTaaaGGTTCA	AGTTCTAAAGGTTCA	Reverse	2567	2581	−7462
						DR2	AGGACAatGGGTAT	AGGACAATGGGTAT	Direct	4413	4426	−5616
						DR1	AGGACAcAAGTCA	AGGACACAAGTCA	Reverse	5535	5547	−4494
						DR3b	[at]G[agc][at][tc][ac]a[at]gAGGTCA	TGATCCAAGAGGTCA	Direct	7198	7212	−2831

Different effects of VD3 in mice and humans have been shown previously for cathelicidin expression 41. The primate (human and chimpanzee) cathelicidin gene promoter contains a consensus VDRE, which is not present in mice, rats, and dogs. The VDRE is suggested to have occurred in a primate progenitor through insertion 42. Similarly, insertions or rearrangements in the mouse TSLP promoter region may have led to functional VDREs, even though the evolutionary advantage of these modifications is unknown. In contrast IL-1 and TNF cytokine responsiveness of TSLP promoter activity is controlled by NF-kB and conserved between human and mouse although there is some discussion which NF-kB site is critical 43,44. Recently, Seok et al. have shown poor correlation between human and murine models with regard to inflammatory responses, indicating marked differences in mechanisms of diseases 45. Comparison of the gene expression profiles during inflammation, the temporal response patterns in several inflammatory diseases, and more specifically the innate immune responses of neutrophils to *Candida albicans* have shown low reproducibility in the current mouse models 45,46.

In conclusion, in contrast to mouse, VD3 does not induce TSLP expression in human and monkey skin. These results are in line with recent studies and epidemiological evidence that VD3 insufficiency rather than elevated VD3 levels may be a contributing factor in the pathogenesis of allergic diseases including AD, asthma, and food allergies 47–50. Although the VD3A-induced AD mouse model is of use in studying an AD-like phenotype in mice, our results indicate that the VD3 dependent mechanisms involved in the induction of TSLP and subsequent eczema in mice do not translate directly to human AD.
