# Supplementary material for: TSLP is differentially regulated by vitamin D3 and cytokines in human skin
Source: Immun Inflamm Dis. 2015 Feb 16;3(1):32–43. doi: 10.1002/iid3.48 (PMC4386913; doi:10.1002/iid3.48)
Supplement: Supplementary file 1 [file iid30003-0032-sd1.docx]

**Supplementary Methods**

**Human primary keratinocytes culture**

Human primary adult keratinocytes were isolated from normal skin as described previously [E1]. Passage 2 keratinocytes were cultured in keratinocyte growth medium (KGM-2) (Lonza Group Ltd., Basel, Switzerland) until 70% confluence and differentiated for three days in the same medium, supplemented with Calcium up to 1,3 mM. Cells were differentiated for two more days in hydrocortisone-depleted KGM-2 with Calcium, since hydrocortisone has been described to decrease TSLP expression [E2,3]. Cells were stimulated with 100 nM calcipotriol (Sigma-Aldrich Co., St Louis, MO) in DMSO (0.1%), DMSO 0.1% (Sigma-Aldrich) or a cytokine mixture containing IL-4 (100 ng/ml), IL-13 (100 ng/ml), and TNF-α (20 ng/ml) (R&D Systems, Minneapolis, MN), as positive control for the induction of TSLP [E4]. A calcipotriol concentration curve was performed at 10, 30, 100 and 300 nM (n=1).

**Normal human skin biopsies culture**

Normal human skin biopsies were obtained as resected material after cosmetic surgery procedures. 4 mm biopsies (n=3) were cultured in Dulbecco’s Modified Eagle Medium (DMEM) containing 10% FBS, 1% pyruvate, 1% HEPES, and 2% penicillin/streptomycin and stimulated with 100 nM calcipotriol, DMSO or a cytokine mixture (see above). A calcipotriol concentration curve was performed at 10, 30, 100 and 300 nM (n=1). To study the effects of a topically applied commercially available VD3 ointment (calcitriol; Silkis®, Galderma, Switzerland), calcitriol ointment or vaseline were applied to a 20 cm^2^ piece of full thickness (including some subcutis) normal human skin (n=3), for 30 minutes at 37°C. 1/3 of the skin surface was left untreated and was used as control. After incubation, 4 mm biopsies were taken and cultured at air-liquid interface in the medium described above on a trans-well system (Greiner Bio-One, Germany) for 24h, 48h, 72h. Culture media level was adjusted to ensure optimal air exposure of the epidermis.

**RNA isolation and Real-time PCR**

**Human material**: Total RNA was isolated from biopsy-derived cryosections (60x20 μm cryosections), using RNeasy Micro kit (Qiagen N.V., Venlo, The Netherlands). RNeasy Mini kit (Qiagen) was used to isolate total RNA from keratinocyte cultures. cDNA synthesis was performed using a BioRad iScript cDNA synthesis kit (BioRad, Hercules, CA). Real-time quantitative PCR was done on a BioRad MyiQ real time PCR Detection System, using iQ SYBR Green Supermix kit (BioRad). Two forms of TSLP mRNA (long and short) have been described in literature, with only the long transcript is translated into a functional protein [E5,6]. A primers set which specifically detects the long form TSLP transcript was designed. The following primers were used: TSLP forward: AGTGGGACCAAAAGTACCGAGTT, TSLP reverse: GGATTGAAGGTTAGGCTCTGG, Cyp24 forward: GGTGACATCTACGGCGTACAC, CYP24 reverse: CTTGAGACCCCCTTTCCAGAG and glyceraldehyde 3-phosphate dehydrogenase (GAPDH) forward: AGAAGGCTGGGGCTCATTT, GAPDH reverse: GAGGCATTGCTGATGATCTTG. The amount of each mRNA was normalised to the amount of GAPDH in the same sample. Relative increases in mRNA expression were calculated using the 2^-∆∆CT method [E7].

**Mouse and monkey material**: Frozen skin samples were homogenized using Cryogenic Tissue Pulverizer (Research Products Int., Mount Prospect, IL). Total RNA was isolated using RNeasy Midi kit (Qiagen, Chatsworth, CA) according to manufacturer instructions. RNA quality was assessed by Agilent Bioanalyzer Nanochip (Agilent Technologies, Santa Clara, CA) analysis to check for intact 28S and 18S ribosomal RNA bands.

Total RNA was treated with DNase I to remove any contaminating genomic DNA (Ambion, Austin, TX), then reverse transcribed into cDNA using a combination of random hexamers and oligo-dT primers (Promega, Madison, WI). Gene expression levels were measured using real-time quantitative PCR and the ABI 7300 Sequence Detection System (Perkin Elmer, Applied Biosystems, Foster City, CA). Primers for human Cyp24 that crossreact to cyno Cyp24 were obtained commercially from Applied Biosystems (Foster City, CA). Real-time PCR amplification of the housekeeping gene ubiquitin was performed for each sample to allow for normalization between samples by the Δ-Δ Ct method (ABI User Bulletin #2, 1997). The equation 1.8 e (Ct of ubiquitin minus Ct of gene being measured) x 104 was used to obtain normalized values. The Δ-Δ Ct method described above results in normalized expression values relative to the housekeeping gene ubiquitin. Normalized values less than 1.0 are considered to be at the limit of detection for this method and were considered to be negative for analysis.

**TSLP Elisa-Monkey**

Cyno TSLP was detected using in-house Assay for Cynomolgus Monkey TSLP Baseline using an electrochemiluminescence (ECL) immunoassay method. Briefly, a sandwich is formed on the MSD plate (Meso Scale Discovery, Rockville, MD) surface between cyno TSLP in the sample and both capture reagent and detection antibody. The capture reagent is a biotinylated rat anti-human TSLP monoclonal antibody GNE01.23B12.H8.A4. A rat anti-NHP TSLP monoclonal antibody, labelled with ruthenium tris-bipyridine chelate Rat anti [TSLP_NHP] JL10.34H11.A8, is used as the detection antibody. This antigen-antibody complex is captured by streptavidin, which is coated on the MSD plate surface. The plate is put into the Meso Scale Discovery SECTOR™ Imager 6000 and a voltage is applied to the bottom of the plate initiating the ECL signal from the label upon electrochemical stimulation at the electrode surface. The resulting signal produced is directly proportional to the concentration of cyno TSLP in the sample.

**References**

E1 Tjabringa G, Bergers M, van RD, de BR, Lamme E, Schalkwijk J. Development and validation of human psoriatic skin equivalents. *Am J Pathol* 2008; 173:815-23.

E2 Le TA, Takai T, Kinoshita H, Suto H, Ikeda S, Okumura K et al. Inhibition of double-stranded RNA-induced TSLP in human keratinocytes by glucocorticoids. *Allergy* 2009; 64:1231-2.

E3 Le TA, Takai T, Vu AT, Kinoshita H, Ikeda S, Ogawa H et al. Glucocorticoids inhibit double-stranded RNA-induced thymic stromal lymphopoietin release from keratinocytes in an atopic cytokine milieu more effectively than tacrolimus. *Int Arch Allergy Immunol* 2010; 153:27-34.

E4 Bogiatzi SI, Fernandez I, Bichet JC, Marloie-Provost MA, Volpe E, Sastre X et al. Cutting Edge: Proinflammatory and Th2 cytokines synergize to induce thymic stromal lymphopoietin production by human skin keratinocytes. *J Immunol* 2007; 178:3373-7.

E5 Harada M, Hirota T, Jodo AI, Hitomi Y, Sakashita M, Tsunoda T et al. Thymic stromal lymphopoietin gene promoter polymorphisms are associated with susceptibility to bronchial asthma. *Am J Respir Cell Mol Biol* 2011; 44:787-93.

E6 Harada M, Hirota T, Jodo AI, Doi S, Kameda M, Fujita K et al. Functional analysis of the thymic stromal lymphopoietin variants in human bronchial epithelial cells. *Am J Respir Cell Mol Biol* 2009; 40:368-74.

E7 Livak KJ, Schmittgen TD. Analysis of relative gene expression data using real-time quantitative PCR and the 2(-Delta Delta C(T)) Method. *Methods* 2001; 25:402-8.
